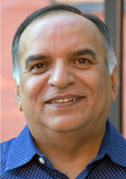# Recent progress of defect chemistry on 2D materials for advanced battery anodes

**DOI:** 10.1002/asia.202000908

**Published:** 2020-09-30

**Authors:** Nabil Khossossi, Deobrat Singh, Abdelmajid Ainane, Rajeev Ahuja

**Affiliations:** ^1^ Condensed Matter Theory Group Materials Theory Division Department of Physics and Astronomy Uppsala University Box 516 75120 Uppsala Sweden; ^2^ Laboratoire de Physique des Matèriaux et Modélisations des Systèmes LP2MS) Faculty of Sciences Department of Physics Moulay Ismail University Meknes Morocco; ^3^ Applied Materials Physics Department of Materials Science and Engineering Royal Institute of Technology (KTH) S-100 44 Stockholm Sweden; ^4^ Condensed Matter Theory Group Materials Theory Division Department of Physics and Astronomy Uppsala University Box 516 75120 Uppsala Sweden

**Keywords:** Defect chemistry, Ultrathin 2D materials, Anode materials for rechargeable batteries, Energy storage, Metal-ion batteries

## Abstract

The rational design of anode materials plays a significant factor in harnessing energy storage. With an in‐depth insight into the relationships and mechanisms that underlie the charge and discharge process of two‐dimensional (2D) anode materials. The efficiency of rechargeable batteries has significantly been improved through the implementation of defect chemistry on anode materials. This mini review highlights the recent progress achieved in defect chemistry on 2D materials for advanced rechargeable battery electrodes, including vacancies, chemical functionalization, grain boundary, Stone Wales defects, holes and cracks, folding and wrinkling, layered von der Waals (vdW) heterostructure in 2D materials. The defect chemistry on 2D materials provides numerous features such as a more active adsorption sites, great adsorption energy, better ions‐diffusion and therefore higher ion storage, which enhances the efficiency of the battery electrode.

## Graphical abstract

Defect chemistry in 2D materials constitutes a major area of research in materials science leading to a new promising anode materials for metal‐ions batteries.

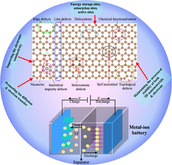

## 1 Introduction

Owing to the widespread consumption of fossil fuels, the two main global challenges facing the world nowadays consist of environmental‐pollution and energy issues. The establishment of new electrochemical‐based energy technologies offers a significant opportunity to mitigate and overcome both the climate change and energy challenges.[^1^, ^2^, ^3^] Nevertheless, in terms of efficiency, price and sustainability, the electric vehicles (EV) are widely depend upon the type of material utilized for the negative electrodes. The development of high‐performance negative electrode materials represents simultaneously a theoretical and an experimental challenge. In addition, given the fact that both the electronic properties with the nature of anode materials have the potential to characterize the charge transfer process as well as the charge/discharge kinetics, a modification and improvement of the electrochemical features can be achieved through the adjustment of the anode material structure. Indeed, 2D materials as well as the defect chemistry constitutes for some years now a relevant approach allowing in particular the modification and improvement of surface properties and electronic structure of 2D materials and was largely applied in anode materials for rechargeable batteries.[^4^, ^5^, ^6^, ^7^, ^8^]

**Figure 1:**
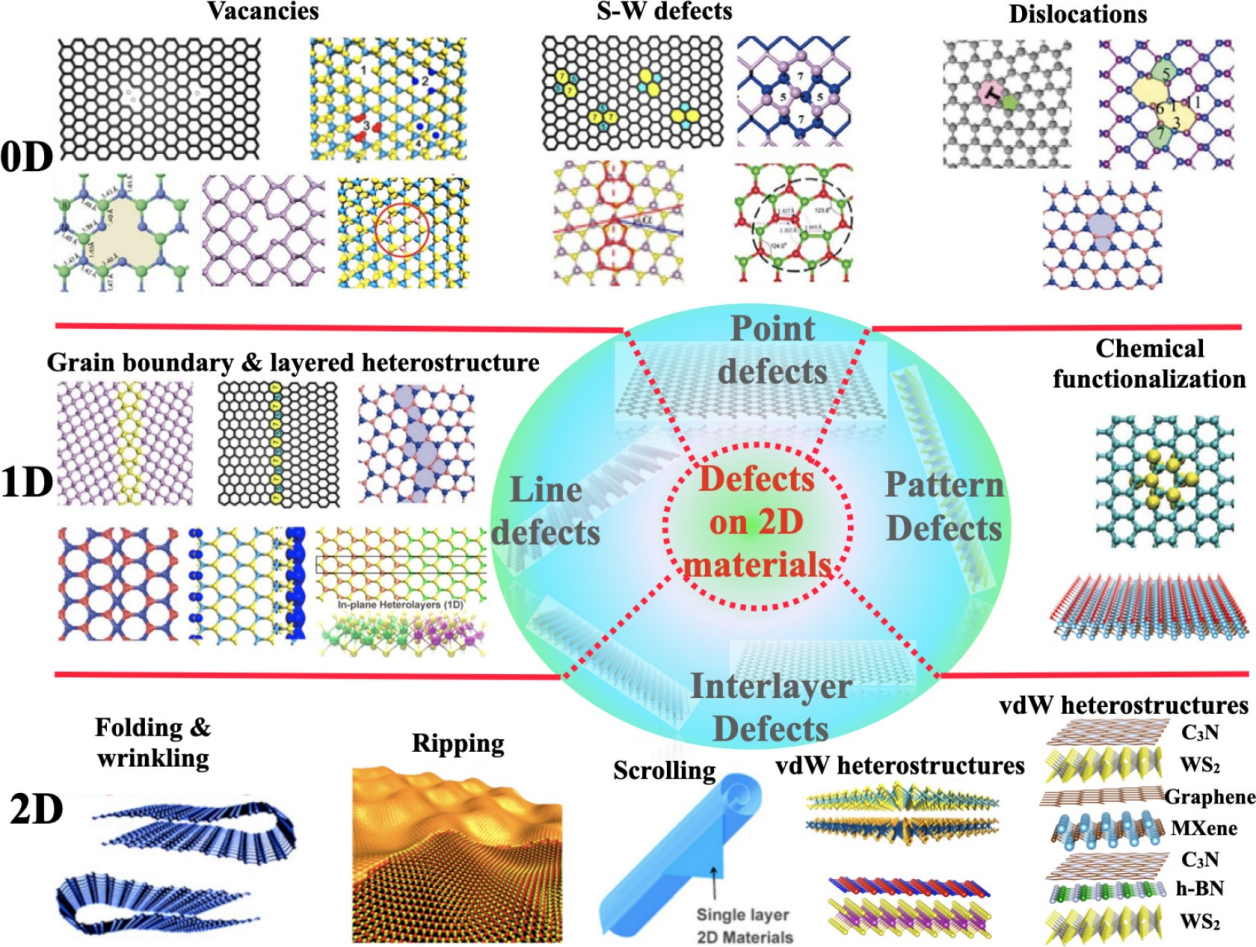
2D materials with various types of defects. Analogous to the case of macroscopic crystalline materials, structural defects in graphene and other 2D materials have different dimensionalities. Such as zero‐dimensional (0D) point defects consist of vacancies, Stone‐Wales (S−W) defects, adatoms, dislocations and substitutions. One‐dimensional (1D) linear defects arise in situations different from those of bulk crystals due to the reduced dimensionality. Not only edge dislocations, but also grain boundaries are 1D lines along which atoms are arranged abnormally. Also, 1D defects such as edges, and phase interfaces. Interstitial between layers can bridge adjacent layers, forming higher dimensional structures. Stacking failure is another typical defect in few‐layer graphene and stacking it with other 2D layered materials. Particular 2D defects such as folding, wrinkled, displacement, ripple and heterostructures stacked vertically. Reprinted with permission from References [18,20, 28–36].

In recent decade, 2D materials namely graphene,[^9^, ^10^] black phosphorene (BP),[^11^, ^12^] hexagonal boron phosphide/nitride/arsenic (h‐BP, h‐BN, and h‐BAs),[^13^, ^14^] transition‐metal dichalcogenide (TMDs) including MS_2_ with M=Mo, W, Ta, Fe, Co, Ni, and Sn,^[15]^ etc. have been widely studied and constitute one of the most interesting category of materials. Due to their outstanding characteristics as well as their suitability for energy storage applications. According to the 2nd law of thermodynamics at equilibrium, The defects chemistry on 2D materials are unavoidable, although, they can also be accidentally or deliberately inserted into crystalline structure of 2D materials which leads to some suitable or non‐suitable impacts on their chemical and physical characteristics.[^16^, ^17^] Among defects chemistry on 2D materials, Zero‐dimensional (0D) point defects consist on vacancy, dislocations, substitution and Stone‐Wales (S−W) defects. One‐dimensional (1D) linear defects consist on grain boundaries in which atoms are arranged abnormally, edge, phase interfaces and the stacking failure in few‐layer material. And finally, 2D defects including folding & wrinkling, ripping & scrolling, and heterostructures stacked vertically, as summarized in Figure 1.[^16^, ^18^, ^19^, ^20^, ^21^] Numerous advances have been made in examining the failure mechanisms underlying defects chemistry on 2D materials. These defects can adopt a variety of atomic configurations based on the differences in the structural and energetic properties (lattice constants and cohesive/ bonding energies, etc.) of 2D materials. Hence, the energetic characteristics of defects chemistry are widely dependent on the structure design, arrangement of atoms and their dimensionality, which can considerably influence the failure characteristics of the 2D monolayers.[^20^, ^21^, ^22^]

Over the past few years, considerable attention has been devoted to improving and developing metal‐ion batteries batteries owing to their ecological character, durability and significant energy‐density performance,[^8^, ^23^, ^24^, ^25^] Nevertheless, several issues hinder their further progress, especially, the limited conductivity, their capacity to store metal ions and their stability within metal‐ion battery. Accordingly, it is necessary to rationally develop a new anode materials that have numerous possible adsorption sites to further enhance their specific capacity and energy density, thus facilitate their implementation in metal‐ion batteries. Being a potent technique, defect chemistry has the potential to provide 2D anode materials with new features, from more active adsorption sites to more rapid metal‐ions diffusion, and more metal‐ions storage, etc.[^25^, ^26^] Furthermore, it is expected that the formation of defects chemistry on 2D materials may enhance the adsorption energy as well as facilitate the electrochemical process transitions.[^22^, ^27^] Hence, it is indispensable to design properly defects chemistry on 2D anode materials to further elucidate the mechanism of these defects on the charge/discharge processes of metal‐ions batteries.

Recognizing all the aforementioned challenges, this mini review aims to offer a detailed insight into recent progress achieved in the area of defect chemistry and their effect on 2D anode materials for rechargeable batteries. We will initially introduce the defect chemistry in 2D materials, and categorize defects chemistry based on their dimensionality. Then, we will describe briefly how these defects can noticeably modify physical and chemical characteristics of 2D materials in Section 2. Subsequently, the latest achievements regarding the defect chemistry on 2D anode materials for metal‐ion batteries will be discussed in details. Specifically, each subsection will highlight the role of each defect chemistry on the 2D anode as well as describe their effects on the electrochemical properties of metals‐ion batteries. Finally, we will present some issues and prospects for the upcoming studies related to defect chemistry and their application on metals‐ion batteries. Through this in‐depth overview, the readers will be able to gain a deeper understanding about the critical role of defect engineering in 2D anode materials as well as suggest improved approaches for expediting the advancement of metal ion batteries.

## 2 Defect chemistry in 2D materials

The defect chemistry within 2D materials has been recognized as a particularly controversial area of research since the first exfoliation of single‐layer graphene in 2004. Many defect chemistry models in the framework of 2D materials have a considerable potential to significantly enhance the functionality of 2D materials over a range of energy storage technologies, particularly metal‐ion batteries, thus the amount of published papers or (mini)‐reviews devoted to different defect chemistry on 2D materials is growing steadily. A brief overview is therefore presented in this section describing the latest research advances related to each category of defects in the chemistry on 2D materials separately. The Figure 1 illustrates the main types of chemical defects expected in pure 2D materials.

### 2.1 *Zero‐Dimensional* defects in 2D materials

Zero‐Dimensional defect represent the most basic and widespread defects chemistry in 2D materials, know also as lattice point defects chemistry. As illustrated in Figure 1, such defects do not exhibit a lattice structure along any direction. They are situated within individual sites of the 2D structure; which may be consistently occupied by certain selected atoms or may be empty sites. Nowadays, this concept has become an indispensable aspect in both physical and chemical characteristics of 2D materials; from ion‐conductivity to Ions‐diffusion, binding energy, electronic behaviour and many other properties. In accordance with the character of the deviation with respect to pure 2D materials structure, one can distinguish different types of 0D‐defects for 2D materials:

- **Vacancies**: Proceeding from the vacancies, being the most investigated 0D‐defects in 2D materials.[^8^, ^11^, ^37^, ^38^, ^39^, ^40^] They can be occur accidentally during preparation processes or voluntarily through processing. Such defects are formed during molecular statics and dynamics simulations through the stripping of atoms from the 2D structure. The vacancies in 2D materials are typically classified into single vacancies (SV) that require the removal of a single atom in the 2D structure, double vacancies (DV) that involve the suppression of a two adjacent atoms, and multiple vacancies (MV) based on the number of atoms removed in the 2D structure. While discussing vacancies defects, it is noteworthy to underline that a key amount which mainly determines their thermodynamic‐equilibrium concentration and energetic stability is the formation energy. The formation energy of SV in black phosphorene (BP) materials is approximately 1.65 eV, considerably lower compared to that of graphene monolayer with 7.57 eV.^[41]^ This can be attributed to the fact in the case of BP the formation energy is related to the P−P bonds which are intrinsically more flexible than C−C bonds which are much higher, but also to the bending stiffness and thickness of 2D BP materials. In the case of 2D TMDs, i. e. MoS_*S*_ materials, the formation energy of SV (V_*S*_: Sulfur vacancy) ranges between 1.22 eV and 2.25 eV, which is lower and closer to that required for phosphorene. That can be attributed to the high concentration of V_*S*_ commonly obtained in the experiments.[^42^, ^43^] As mentioned above, the double and multiples vacancies which be created either by removing two or more neighboring atoms in graphene and graphene like‐structure or by the coalescence of two or more SVs. For DVs, it can be replicated and reflected in the formation of two pentagons and one octagon like in the case of V_2_(5‐8‐5) defect. for other type of materials such as TMDs (MoS_2_), once V_*Mo*_ is created, the sulfur atoms surrounding it have a strong propensity to lose. Therefore, the reason why V_*Mo*_ is not considered as a stand alone, and that most of the V_*Mo*_ vacancies appear as defect complexes MoS3 vacancies.^[43]^
- **Stone‐Wales defects**: One of the outstanding features of 2D materials exhibiting a hexagonal or honeycomb geometry consists in the possibility of forming non‐hexagonal rings through the rotation of the local bonds as shown in Figure 1(0D). This new category of 0D‐defects in 2D materials are constituted independently of any adding or removing of atoms on the pure 2D structure. Known also as topological defects, and they are the widely explored in the case of single layer graphene.[^44^, ^45^, ^46^, ^47^, ^48^] As shown in Figure 1(0D) of S−W defect in the case of graphene monolayer, it can be obtained by rotating a pair of neighbouring C‐atoms by 90 ° around the mid‐point of the linking bond. This type of defects chemistry also occur in other 2D materials beyond graphene for instance h‐BN, TMDs MoS_2_, and BP[^11^, ^49^, ^50^] (see Figure 1(0D). From the energetic stability point of view, the formation energy of a S−W defect within the h‐BN monolayer is greater as compared to that of graphene, whereas the lowest formation energy is found in BP owing to its unique out‐of‐plane buckling geometry structure.[^51^, ^52^]
- **Dislocations defects**: represent a different class of 0D‐defect, susceptible to be formed through CVD growth, electron beam sputtering (EBS) or through a mixture of other 0D‐defects.^[53]^ This type of defects generally have a significant effect on physical characteristics of 2D materials. The dislocation defects are illustrated in the case of graphene as pentagon/heptagon 5–7 paires, and is represented in the case of h‐BN as 5–7 and 4–8 paires, while in the case of BP, both dislocations 5–7 and 4–8 occur as a consequence of an‐isotropic buckled lattice structure.[^42^, ^54^] It is also important to notice that the formation of dislocation defects considerably affects the characteristics of graphene and beyond graphene like structure. For example, 5–7 pairs are susceptible to improve or reduce the stability of the graphene as a function of the concentration of defects.^[55]^

### 2.2 *One‐Dimensional* defects in 2D materials

One‐dimensional defect chemistry generally covering edges, phase‐interfaces, and nanowires (NW). We will mainly concentrate in this section on two main categories, namely line defects and patern defects in 2D materials owing to their significant promise in various applications, including energy storage (see Figure 1(1D)).

- **Line defects**: Grain boundary represents a largely studied defects of line defects chemistry in 2D materials. Wide‐scale single layer graphene produced through CVD method is composed by monocrystalline grains of various lattice‐orientations. A Grain boundary defects chemistry in 2D materials can be constituted through a range of dislocations‐defects disposed in a linear arrangement. Basically, the grain boundaries were categorized according to the angle of inclination which divides two neighboring grains, The first one, the small angle grain boundaries in which the dislocations is spaced with a relatively great distance, whereas in large angle grain boundaries, the dislocations is narrowly spaced and may overlap at times.[^56^, ^57^, ^58^] Layered heterostructure constitute also another kind of line defect. Some lateral heterostructures have been reported in the recent years including MoX_2_‐WX_2_, MoY_2_/WY_2_, MoX_2_/MoY_2_ and WX_2_‐WY_2_ heterostructures, with X=S and Y=Se.[^36^, ^59^, ^60^]
- **Chemical functionalization**: Represent one of pattern defects in 2D materials, it is likewise one of the principal factors for analyzing the interaction behavior of 2D materials with their surroundings.[^61^, ^62^, ^63^] Several sets of chemical substances such as Hydrogen (H2), Sulfur (S), Chlorine (Cl), Bormine (Br), Fluorine (F) and Oxygen (O) may be fixed upon the surface of 2D materials for the purpose of modulating their geometry structures as well as their physical and chemical properties.[^64^, ^65^] The chemical functionalization of 2D materials has become indispensable for a wide variety of new energy storage applications, including hydrogen storage, gas sensing, supercapacitors, and rechargeable batteris electrodes.[^14^, ^66^, ^67^, ^68^, ^69^, ^70^] One can distinguish two essential forms of chemical functionalization: the first one, known as covalent‐functionalization, consist in the creation of covalent bonds, and the second one, non‐covalent functionalization involving the physisorption or physical‐adsorption of molecules on the surface of 2D structure. The first one leads to a significant change on the geometry as well as in the characteristics of 2D materials. For 2D materials with graphene like structure, the chemical‐functionalization corresponds to the rehybridization of sp^2^‐configurations to sp^3^‐configurations. The covalent‐functionalization modulation of 2D materials may be obtained through various approaches such as Nucleophilic‐Substitution (NS) and condensation technique, etc..[^14^, ^67^, ^71^]

### 2.3 *Two‐Dimensional* defects in 2D materials

**Figure 2:**
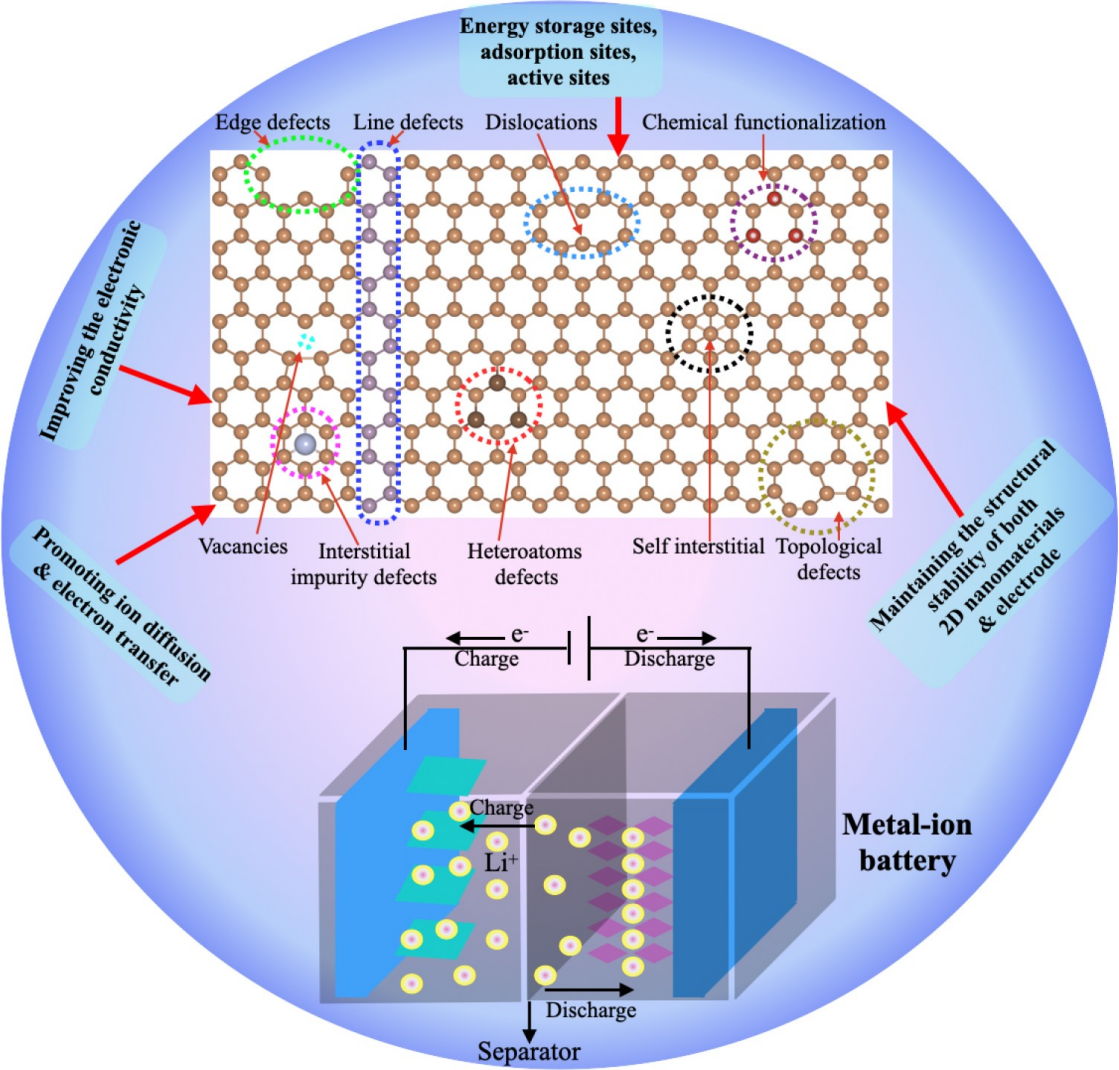
The schematic representation of various defects in 2D materials for energy storage in metal‐ion batteries.

The 2D‐defect chemistry that is typical for vdW and inter‐layer materials, including holes‐craks, folding‐wrinkling, ripping, and vdW‐heterostructure, can also occur in 2D materials, although these will not be covered in this mini‐review, however, a few defect type related in interlayer materials are significant and will be outlined shortly. First, the holes within 2D materials which constitute a kind of geometrical defect susceptible to be constituted either by a certain number of vacancies, or by electron‐beam irradiation (EBI) through atomic clusters. After formation of a hole, it expands constantly through EBI method. In the case of single‐layer graphene and graphene like structure, such defects chemistry present both atomic structures as well as 5–7 pairs of zig‐zag structures.[^72^, ^73^] A further defect is cracking, which can occur in 2D materials through the creation of pores. Such defects typically can appear in a variety of sizes/orientations, and are often critical in the failure of 2D materials. In addition, The adjacent layers of 2D materials are connected through van der Waals‐type interactions, the strength of vdW‐interactions is highly dependent on the spacing between the different layers, which is related to the accumulation of many layer 2D structures. The vdW‐interface strongly affects the chemical and physical characteristics of fewlayred 2D materials, this vdW‐interface coupled with stacking and layer alignment is considered as a 2D defect as illustrated in Figure 2(2D).

For an in‐depth understanding the effective ways of introducing the above‐mentioned defects chemistry in 2D materials, three advanced characterization ways have been successfully exploited widely in recent years, namely the transmission‐electron microscope (TEM), Scanning‐Tunneling Microscope (STM) and X‐ray Photo‐electron Spectroscopy (XPS). Despite the fact that both effective ways TEM and STM provide a structural image at the atomic‐scale, they encounter a big challenges of complicated sample set‐up and limited inspection surfaces.[^74^, ^75^, ^76^, ^77^, ^78^, ^79^] On the other hand, a statistical process based on X‐ray Photo‐electron Spectroscopy presents the disadvantage of a limited area resolution.^[80]^ In contrast, the optical spectroscopic techniques, especially Raman and photoluminescence spectroscopies, provide an efficient and nondestructive way to characterize defect on 2D materials. With these two effective ways, all the information on 2D materials structure, including the electronic properties and lattice‐vibrations can be extracted and exploited to survey the thickness in the case of monolayer and number of layers in the case of vdW heterostructures, bi‐axial and uni‐axial strain, structural stability of the materials, and more especially the defects chemistry as well as a stacking orders of 2D materials.[^81^, ^82^, ^83^, ^84^, ^85^, ^86^, ^87^] Indeed, it has been recognized that Raman‐spectroscopy constitutes a potential and effective way to characterize defects chemistry in 2D materials with graphene or graphene like‐structure, owing to the existence of D‐ and D’‐peaks relative to defects in the spectrum of samples.[^88^, ^89^, ^90^, ^91^] In the case of other 2D materials, namly, TMDs, there also appear some additional Raman‐peaks immediately after introducing the defects chemistry with an intensity correlated to the density of the defects.[^92^, ^93^]

In summary, The defect chemistry in 2D materials leads to some perturbation of adjacent atoms as well as lattice mismatch, leading to efficient regulation of the structural/electronic structure and physical/chemical properties of 2D materials which will more strongly affect the electrochemical characteristics of the rechargeable battery anode materials. For example, it has been shown by Wang et al.^[94]^ that the insertion of oxygen defects can adequately control the valence‐state of metal‐ions in TMO materials, thereby modifying its electronic features as well as its catalytic efficiency. Furthermore, they controllably generated defects on the structure of high‐oriented graphite, and showed also that it is possible to induce a surface charge on the defected sites. In addition, It has been found by Dai et al. that the hetero‐atoms with varying electro‐negativity when exchanged for one C‐atom may result the modification of electro‐chemical characteristics of graphene monolayer.^[95]^ Defect engineering plays a major role with a beneficial effect upon the physical/chemical features of materials and their electrohcemical performance when used for energy storage systems.

## 3 Defective 2D anode materials for rechargeable Batteries

There are still some challenges to the direct use of 2D nanolayererd structures in energy storage devices. Pristine graphene, with higher edge activity than surface activity, has a lower chemical affinity than other nanostructured materials, resulting in poor interface compatibility, long diffusion pathways, and slow chemical reactivity. In case of lithium ion storage, it also suffers from large irreversible capacity, low initial Coulombic efficiency and rapid fading of capacity, in addition to high hysteresis between discharge and charge curves as a result of its intrinsic framework structure and a strong tendency to re‐stack during the battery assembly. In the matter of layered transition metal dichalcogendies (TMD)/transition metal oxides (TMO) nanolayered structures, repeated insertion/extraction of Li^+^ ions often results in dramatic variations in layer spacing, along with successive phase transitions. To address those challenges, edge/surface functionalization of 2D nanolayered structures has been shown to be effective in modifying many physical properties, including intrinsic conductivity and electronic band structure.^[96]^ For high performance lithium ions batteries (LIB) applications, this strategy is primarily accomplished by introducing or removing certain atoms, ions, or bonds to enhance their electronic properties, interface structures, or chemical activities to accommodate repeated intercalation/deintercalation of lithium and other metal ions during processes.

### 3.1 Types of defects used in metal‐ion batteries (MIBs)

The existence of rich‐defects such as 0D defects (point defects includes vacancies, S−W defects, dislocations, etc.), 1D defects (grain boundary and layered heterostructure), 2D defects (interlayer defects includes vdW heterostructure, ripping, scrolling, folding and wrinkling, etc.) and pattern defects includes different types of surface and chemical functionalizations could lead to vacancies or stress in the basal planes of 2D nanosheets and, therefore, dramatically increase the rates of exposure of active sites, which would greatly increase their capacity to accommodate the intercalation and diffusion of Li‐ions. Moreover, benifits of these structural defects in 2D nanosheets significantly improving the electrical conductivity. These types of defects in 2D nanosheets are mainly synthesized by two types of approches, i) direct growth via chemical vapor transport (CVT) and ii) CVD methods along with some post‐treatments such as electron/ion irradiation, plasma treatments and annealed at high temperature under different gases.^[20]^ In this review article, various types of defects that are often adopted for engineering defects are summarized in the following sections.

### 3.2 Doping defects for MIBs

The doping of active atoms or ions in graphene and other 2D nanolayered structures mainly requires to improve their electrochemical properties as electrode materials for LIBs and other metal ions batteries. The heteroatom doped graphene is a significantly common and specific category. To overcome the structural limitations of the skeleton of graphene, some heteroatoms, for examples F, Cl, Br, S, P, N, B, etc., are introduced under graphene with the expectation of modifying the energy of surface adsorption, reducing the ion diffusion barrier and consequently improving battery performance. In addition, these doped graphene‐based nanostructured materials could offer a higher theoretical capacity compared with pristine graphene, due to the synergistic storage mechanism with heteroatoms. Generally, synergistic effect appears with the combination of crystalline materials and metal nanoparticles.^[97]^ The synergistic effect is activated to accelerate the charge transfer and promote the catalytic activity to alter the electronic structure by multimetal doping strategy.^[98]^ According to Liao et al., the effect of edge doping of B, C, N and O on zigzag graphene nanoribons and found that the maximum charge/carrier charge in graphene obeys a rule of [8‐(n+1)], where n is the valence electron number of the atom at the edge site constituting the adsorption site.[^99^, ^100^] If metal atoms (Cr, Mn, Fe, etc.) were used as doping elements, graphene could also be bent again to form metal‐welded CNTs with improved electrochemical properties.^[101]^

It is well known that the most electronegative element in the periodic table is fluorine (3.98) which is ≈1.6 times larger than carbon (2.55). In addition, the C−F bond is the strongest covalent bond. Consequently, fluorine‐doped graphene nanolayered structures could offer the highest charge polarization to improve the electrochemical activity and superior stability of the electrode. In the previously investigated results shows that the liquid peeling and dry milling methods gives high‐quality fluorine‐doped graphene.[^102^, ^103^, ^104^] Unlike liquid graphite peel in graphene nanosheets, most fluorine‐doped graphite cannot be easily exfoliated in common solvents, for examples N, N‐dimethylformamide and N‐methyl‐pyrrolidone, under the same conditions. The ultrasonic treatment of fluorine‐doped graphite in 2‐isopropanol solvent (IPA) at room temperature was used to find fluorine‐doped graphene.^[102]^ The final results shows with specific surface area of 125 m^2^ g^−1^, as well as a thickness of approximately 10 nm and a high fluorine content of 49.7%. According to Zhao et al.,^[102]^ the fluorinated graphene nanosheet display high initial reversible capacity of 843 mAh g^−1^ for LIBs and after 50 cycles it maintain reversible capacity of 780 mAh g^−1^. Additionally, the reversible capacity of graphene doped with fluorine was 626 and 336 mAh g^−1^ at high current densities of 0.1 and 0.5 A g^−1^, which is relatively higher than flurinated graphite of 218 and 108 mAh g^−1^ at similar current rate, respevtively.

Other group successfully obtained the flurinated graphite by liquid exfoliation methods in acetonitrile (ACN) and chloroform solvents.^[103]^ Due to the presence of low boiling points of chosen solvents, there is no reduction of C−F bonds at high temperature and pressure. The flurinated graphite were used as electrode materials for the LIBs with a high specific capacity of 775 mAh g^−1^ at a rate of 0.05 C and also it has high‐rate capability and high discharge voltage.^[103]^ For this technique, the selection of appropriate solvents for effective exfoliation is crucial; however, adequate solvents are extremely limited, which has greatly hindered progress towards large‐scale production. In contrast, the low‐cost, simple dry ball milling technique can avoid the use of harmful organic solvents and has great potential for expansion.

In addition, similar to edge‐fluorinated graphene, other edge‐halogenated graphene nanoplates for examples XGnPs where X=Cl, Br, I, N, S, H, etc. has also been successfully synthesized through via dry‐milling graphite in the presence of the doping element, including chlorine, bromine, iodine, nitrogen, sulfur, hydrogen, etc..[^104^, ^105^, ^106^, ^107^, ^108^] In case of FGnPs system shows specific capacity of 650.3 mAh g^−1^ at 0.5 C in the voltage range of 0.02–3 V which is relatively higher than HGnPs system with specific capacity of 511.3 mAh g^−1^ at same voltage regime.^[108]^ In addition after 500 cycles, it maintain higher specific capacity of 498.2 mAh g^−1^ and 208.5 mAh g^−1^ with coulombic efficiency of 76.6% and 40.8% for FGnPs and HGnPs system respectively. It means that the better cycling performance for FGnPs is much better than that of HGnPs system.^[108]^ Xu and co‐workers reported that the HGnP, ClGnP, BrGnP, and IGnP system shows discharge capacities of 1666.9, 1783.6, 1690.4, and 1750.3 mAh g^−1^ in the voltage range of 0.02–3.0 V, respectively. In these reported four system, edge functionalized by iodine showed a higher rate capacity than the others and also it sustain the discharge capacity of 464.1 mAh g^−1^ after 500 cycles. According to that the IGnP system is a great potential as a promising anode material for high energy LIBs.^[106]^

Apart from this, nitrogen atoms were also embedded in the graphene framework to form nitrogen‐doped graphene (NG) nanosheets. Implantation of N‐atoms notably modified the electronic properties of graphene, delivers more active sites, enhances interactions between Li and C ions, and therefore improves Li diffusion and transfer kinetics during LIBs application. It was seen that the growth of NG nanosheets is controlled by using a chemical vapor deposition technique based on liquid precursor.^[127]^ The NG nanosheets provide the reversible capacity of 0.05 mAh cm^−2^ which is relatively higher than that of pristine graphene 0.03 mAh cm^−2^ in the voltage range of 0.02–3.2 V at current density of 5 μAh cm^−2^.^[127]^ Another group successfully synthesized the NG nanosheets by heat treatment of graphite oxide under ammonia for two hours at 800 °C.^[128]^ According to Wang et al.,^[128]^ the NG nanosheets display the charge and discharge capacity of 900 and 250 mAh g^−1^ at a current density of 42 and 2100 mA g^−1^, respectively. Huang et al.,^[129]^ reported that the graphene nanosheets co‐doped with nitrogen and fluorine exhibited the discharge capacity of 1075 mAh g^−1^ at the current density of 0.1 A g^−1^ and also it has high retention capacity of 95% after 2000 cycles at the rate of 5 A g^−1^.

Additionally, different ion‐doped TMO nanosheets were also investigated as an electrode materials for high performance LIBs. It was seen that the intercalations of some cations for examples Na^+^, K^+^, V^4+^, Sn^2+^ etc. in the TMOs nanosheets significantly enhance the electrical conductivity, improving the diffusion of lithium ions and thus improving electrochemical storage performance.^[141]^ Moreover, intercalations of these cations in TMOs naosheets is useful in increasing the inter‐layer spacing to make ion insertion more effective. Lu and co‐workers reported that the TMO nanosheets displayed the specific capcities of 118, 137, 155, 97 and 87 mAh g^−1^ for LiMO, NaMO, KMO, MgMO and CoMO nanostructures at the current density of 30 mA g^−1^, respectively.^[130]^ It was also noticed that the rate capability of these TMOs nanostructures have 61, 50, 58, 48 and 45% for KMO, LiMO, NaMO, MgMO and CoMO nanosheets. These studied system divalent cations (Co^2+^), monovalent cations, including Li^+^, Na^+^ and K^+^ were more favorable for improving the performance of lithium ion storage.^[130]^

### 3.3 S−W defects for MIBs

**Figure 3:**
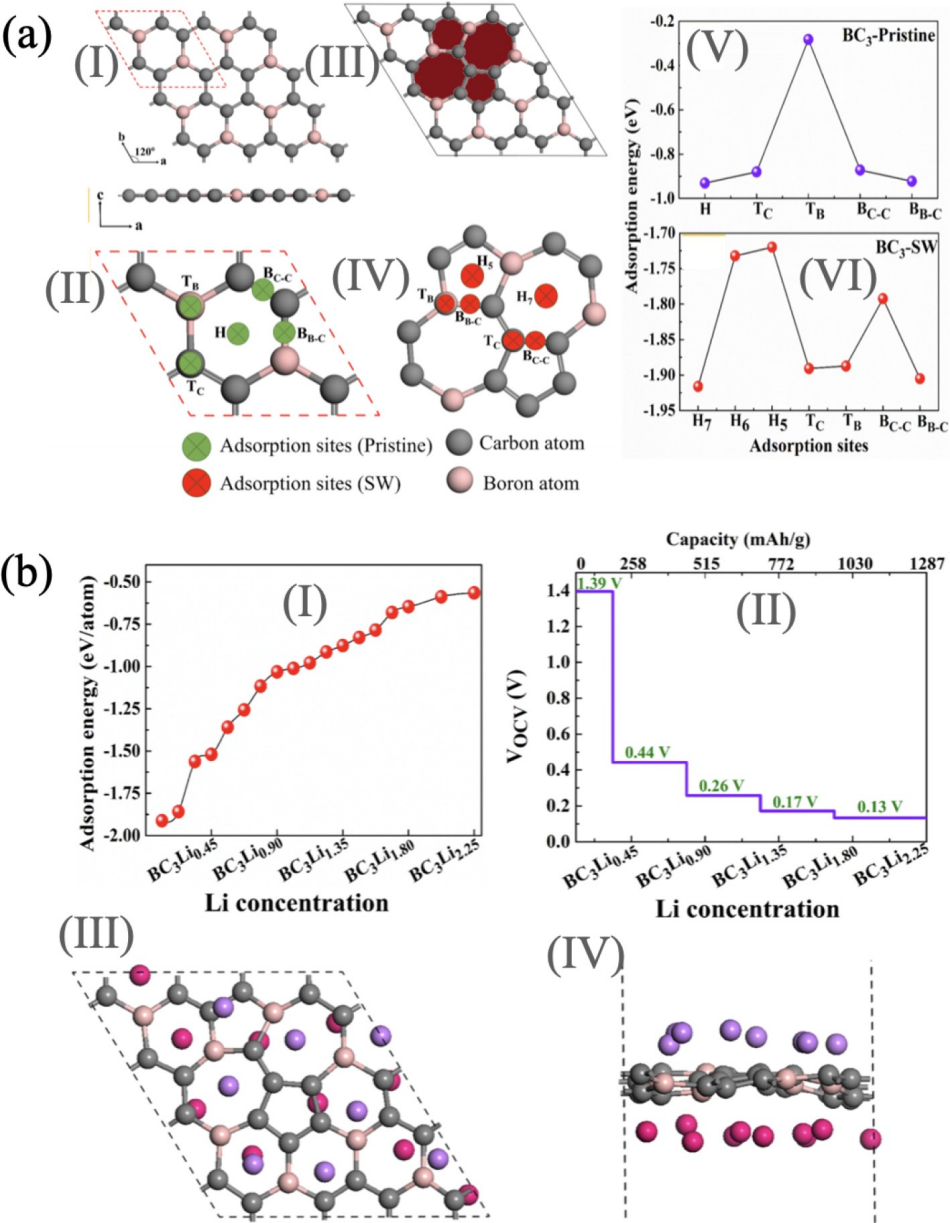
(a) Theoretical prediction of SW defect in BC_3_ monolayer. (I) Pristine BC_3_ with a 2×2 supercell, (II) green circle represents the Li adsorption sites on the BC_3_ monolayer sheet, (III) SW defect in BC_3_ monolayer, (IV) representation of adsorption sites with red circle, (V) adsorption energy at various adsorption sites of pristine BC_3_ monolayer sheet and (VI) adsorption energy of SW defected BC_3_ monolayer. (b) (I) Adsorption energy as a function of Li concentration in a BC_3_ monolayer with a SW defect, (II) variation of voltage profile at maximum intercalation capacity of Li ions on the BC_3_ monolayer with a SW defect, (III) top and (IV) side view of the fully lithiated BC_3_ monolayer with a SW defect from AIMD simulations at 300 K. Reprinted with permission from Ref. [136].

From the theoretical investigations, it was seen that the SW defects in 2D materials significantly alter the electronic properties such as fast charge transfer and display most active sites for metal‐ions adsorptions.[^136^, ^142^, ^143^, ^144^, ^145^] Recently it was reported that the SW defect significantly enhanced the electrochemical performance of Li‐ion battery.^[136]^ Figure 3 a (I–IV) shows the BC_3_ monolayer with pristine and SW defected sheet. When the Li‐atom located at the top of the pristine BC_3_ surface then the adsorption energy are varies from ≈−0.3 eV to −0.90 eV. While Li‐atom placed at the SW defected BC_3_ monolayer sheet, the strength of adsorption energy significantly enhanced (see Figure 3 a(V, VI)). It means that the defected surface are more reactive for metal‐ions battery. Moreover the concentrations of Li‐atoms increases then SW defected BC_3_ sheet have significant strength of adsorption energy for Li‐atoms (see Figure 3 b(I)). Additionally, (see Figure 3 b(II)) shows the variation of voltage profile at different concentration of Li‐ions on the surface of SW defected BC_3_ sheet. Also defected BC_3_ sheet exhibit thermally stable at 300 K ((see Figure 3 b(III, IV)). Most importantly, the SW defected BC_3_ monolayer displayed fast diffusion process for Li‐ion which has almost same and low activation barriers of 0.34 eV and 0.33 eV for pristine and defected BC_3_ monolayer, respectively. Also, it displayed a average open circuit voltage of 0.485 and 0.465 V for pristine and SW defect cases when Li‐ion intercalated on BC_3_ monolayer, respectively. The high theoretical specific capacity are found to be 1144 and 1287 mAh g^−1^ for pristine and SW defected BC_3_ monolayer, respectively. From these investigation we can say that the SW defected BC_3_ monolayer have promising candidates for metal‐ion battery.

Another theoretical work reported that the vacancy and SW defected graphene notably enhance the performance of metal‐ion battery.^[145]^ It was found that the defective graphene sheet energetically stable and enhanced the adsorption and charge transfer between sheet and adsorbed atoms. It was reported that the di‐vacancy defect in graphene shows the specific capacity of 1459 mAh g^−1^ and 2900 mAh g^−1^ for Na‐ion and Ca‐ion battery, respectively. While SW defected graphene have 1071 mAh g^−1^ and 2142 mAh g^−1^ for the Na and Ca‐ion battery, respectively. From these reported high specific capacity will be better anode materials for superior cycling performance for metal‐ions batteries.

### 3.4 Vacancies defects for MIBs

Oxygen vacant hydrogenated TMOs as a vital representative of defect functionalized 2D nanolayered materials have received much attention because of their improved structural stability, electrical conductivity, and kinetics of electrochemical reaction to lithium ion diffusion.[^131^, ^146^, ^147^, ^148^] The hydrogenated *V*_2_ *O*_5_ (H‐*V*_2_ *O*_5_) nanosheets synthesized by Peng et. al.^[131]^ with most of the oxygen vacancies at the O(II) sites by treating *V*_2_ *O*_5_ nanosheets in a H_2_ atmosphere at 200 °C. The H‐*V*_2_ *O*_5_ nanosheets exhibited a discharge capacity of 259 mAh g^−1^ during the first cycle at the current density of 0.1 A g^−1^ and also it was seen that it capacity retained 55% when rate was increased bt 20 times to 2 A g^−1^ with the range of voltage 2–4 V. Moreover after 30 cycles, it is observed that the decay of capacity was 0.05% per cycle. This defective H‐*V*_2_ *O*_5_ nanosheets shows better performance for Li‐ions diffusion and storage.^[131]^

**Figure 4:**
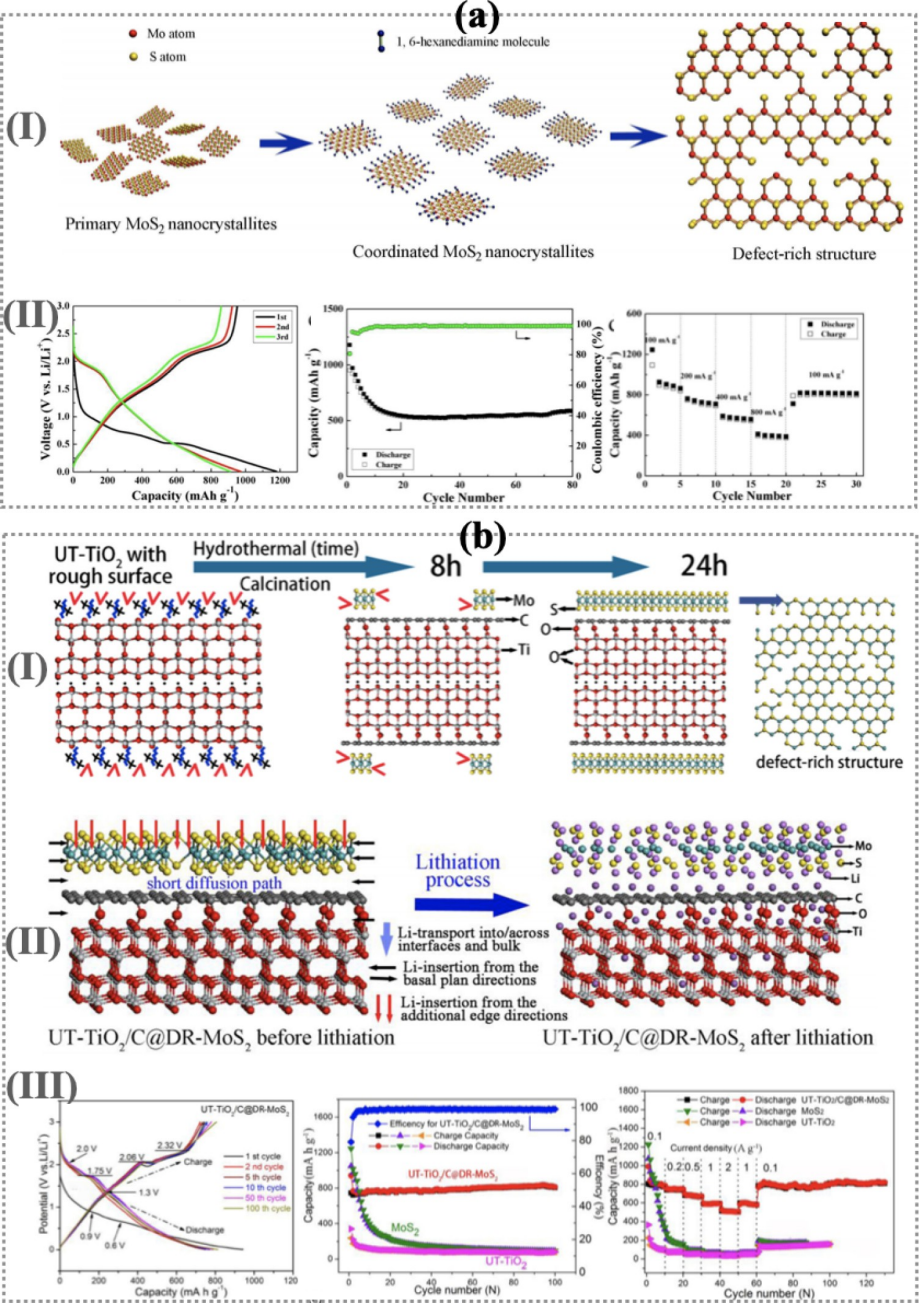
a(I) The Schematic representation to find the defect‐rich MoS_2_ nanosheet, a(II) initial charge and discharge potential profile, cycling performance and corresponding Coulombic efficiency, rate capability of charge and discharge at different current densities for defect‐rich MoS_2_ ultrathin nanosheet. Reproduced with permission from Ref. [132]. b(I) Schematic pathways of the fabrication of hybrid structures of 2D carbon ultra thin MoS_2_ with nano‐sheets TiO_2_ (UT‐TiO_2_/C@DR‐MoS_2_), b(II) schematic representations of the lithiation process of UT‐TiO_2_/C@DR‐MoS_2_ hybrid structures in the first cycle, b(III) charge/discharge voltage profiles of the hybrid structures UT‐TiO_2_/C@DR‐MoS_2_ at a current rate of 0.1 A g^−1^, cycling performance and rate‐capability performance for pristine MoS_2_, UT‐TiO_2_ and UT‐TiO_2_/C@DR‐MoS_2_ hybrid structure. Reproduced with permission from Ref. [149].

Some experimental researchers reported that the defect‐rich TMDs nanostructure materials mainly MoS_2_ ultrathin nanosheets displayed as a high performance of electrode materials. The native defect in MoS_2_ nanosheets with common structural defect for examples molybdenum vacancies (V_*Mo*_), sulfur vacancies (V_*S*_) and molybdenum interstitials (Mo_*i*_), sulfur interstitials (S_*i*_) can significantly change the electronic properties by modifying the density of charge carriers and mobility.[^150^, ^151^, ^152^] The defect‐rich MoS_2_ nanosheets was successfully synthesized (see Figure 4a(I)) by hydrothermal method with the stoichiometric ratio of Mo(VI) and L‐cysteine using 1,6‐hexanediamine.^[132]^ The prepared ultrathin nanosheets was 8–9 nm thick which is approximatly equals to 13–15 sandwiched of S−Mo−S layers. The variations of voltage profile for charge/discharge curves during the lithiations of defect‐rich MoS_2_ untrathin nanosheets electrode as shown in Figure 4a(II). During the lithiation process, the initial discharge/charge capacity of 1179 mAh g^−1^ and 952 mAh g^−1^ with high initial Coulombic efficiency of 81% for defect‐rich MoS_2_ ultrathin nanosheets (see Figure 4b(II)). The reversible discharge capacity could be sustained at 589 mAh g^−1^ at the current density of 100 A g^−1^ after 80 cycles^[132]^ when applied it as a anode for LIBs. And also Coulombic efficiency of 94% which is significantly enhanced after the first cycles. From these results we conclude that the performance of Li‐ions storage is significantly enhanced for superior lithium‐ion battery electrode by intriguing defect‐rich MoS_2_ based nanostructured materials. It was also reported that the reversible capacity of 412 mAh g^−1^ can be achieved for the electrode at a high current density of 800 mA g^−1^ after 15 cycles at different current densities.^[132]^

### 3.5 Interlayer defects for MIBs

The hybridization of nanomaterials with complementary properties in multifunctional nanocomposites that exhibit good synergistic effects in combination with the merits of each constituent is one of the most used strategies to enhance the electrochemical performances of 2D nanomaterials. The excellent properties of 2D nanomaterials in their pristine forms are, however, reported to be insufficient to meet the increasing demands of LIBs applications. It is reported that the pristine graphene have exceptional electron mobility which is chemically active at the edges while not at the surface and which cannot provide a stable potential output, but transition metal oxides (TMO) and transition metal dichalcogenides (TMDs) are inherently inferior in terms of electrical conductivity. It was seen that the most of the TMO, TMDs and other semiconducting 2d nanomaterials hybridized with conductive nanomaterials such as graphene then overall hybrid nanomaterials significantly enhanced the electrical conductivity[^153^, ^154^, ^155^, ^156^, ^157^, ^158^, ^159^] and contributing to suppressing the interior resistance which is very beneficial for electrochemical reaction rate. In the present section, we mainly focus on the hybrid nanomaterials (i. e. interfacial interaction of 2D/2D, 2D/1D and 2D/0D nanomaterials) to see the electrochemical performance of metal‐ions batteries.

Furthermore, Chen and co‐workers sucessfully design the hybrid nanostructures materials sand‐witch of 2D carbon ultra thin MoS_2_ with nano‐sheets TiO_2_ (UT‐TiO_2_/C@DR‐MoS_2_) using a glucose‐assisted hydrothermal reaction at 200 °C.^[149]^ As illustrated in Figure 4b(I) display the complete formation of defect‐rich MoS_2_ ultrathin nanosheets. The representations of molecular structures design for Li atoms insection process to display the lithium storage mechanism (see Figure 4b(II)). When hybrid nanosheets considered as an anode materials for LIB, they performed the superior discharge capacity of 785.9, 585.6, 507.6, and 792.3 mAh g^−1^ at the rate capacity of 0.1, 1.0, 2.0, and 0.1 A g^−1^, respectively and which shows excellent cycling performance of 805.3 mAh g^−1^ after 100 cycles at the rate capacity of 0.1 A g^−1^. During the first discharge step, the defect‐rich MoS_2_ with sufficient edge sites could shorten diffusion paths and effort sufficient diffusion channels. However, after the first discharge step, the MoS_2_ nanostructure would decompose into small nanoparticles and further transform into smaller Mo and S amorphous nanoclusters that would deposit on the TiO_2_@C nanostructure. Table 1 summarized the lists of defected 2D nanomaterials for rechargeable metal‐ions batteries. This breakdown is support in enhancing the contact area between the electrode materials and the electrolyte, and to provide more channels for the diffusion of Li‐ions, thus improving the kinetics of lithiation.^[149]^ Recently a very fascinating work reported by Fang et al.,^[160]^ the defective TiO_2_@reduced graphene oxide (M‐TiO_2_@rGO) displayed the a capacity of 177.1 mAh g^−1^ with the Coulombic efficiency of 74% at 500 mA g^−1^ after 200 cycles for Na‐ion battery. Moreover, M‐TiO_2_@rGO exhibits an energy retention of 84.7% after 10000 cycles. Moreover, Liu et al.^[161]^ reported that the defective TiS_2_ nanosheets have better capacity retention as compared to its pristine nanosheets for metal‐ion battery.

**Table 1:** Lists of the materials, types of defect, fabrication methods and electrochemical performances of defective two‐dimensional nanolayered structures as an electrodes material for metal‐ions batteries.

Type of	Materials	Types of defect	Methods	Capacity	Ref.
battery				[mAh g^−1^]	
Li‐ion	N‐doped hard carbons	Heteroatoms doping	Graphitization process	175	[109]
Li‐ion	MnO−Vo hexagonal sheets	Oxygen vacancies	Thermal reduction	1228	[110]
Li‐ion	Ultrathin Bi_2_MoO_6_ sheets	Oxygen vacancies	Wet‐chemical method	903	[111]
Li‐ion	Li_4_Ti_5_O_12_ nanosheets	Oxygen vacancies	Plasma technology	173	[26]
Li‐ion	SnS_2_/SnO nanosheets	Sulfur vacancies	Plasma technology	1496	[112]
Na‐ion	R‐TiO_2–*x*_ ‐S	Heteroatoms doping	Plasma technology	≈265	[113]
Na‐ion	NC@MoS_2_‐VS	Sulfur vacancies	Thermal reduction	495	[114]
Na‐ion	Soft carbon nanosheets	Micropores and edge defects	Microwave exfoliation	103	[115]
Na‐ion	HMF‐MoS_2_	Cation vacancies	Acid etching	384	[116]
Na‐ion	Ti_0.87_O_2_ nanosheet	Cation vacancies	Chemical exfoliation	490	[117]
Na‐ion	MoS_2_/graphene nanosheets	Intrinsic defects of carbon	Ball‐milling and exfoliation	201	[118]
Mg‐ion	B‐TiO_2–*x*_ nanoflakes	Oxygen vacancies	Atomic substitution	150	[119]
Zn‐ion	Mo/Ti:WO_3_ (MTWO)	Cation vacancies	Wet‐chemical doping	260	[120]
Zn‐ion	ZnMn_2_O_4_ spinel	Cation vacancies	Chemical method	150	[121]
Zn‐ion	Oxygen‐deficient MnO_2_ nanosheets	Oxygen vacancies	Wet‐chemical method	345	[122]
K‐ion	N‐doped hollow carbon	N‐doping and porous	Thermal treatment	≈294	[123]
K‐ion	Graphitic nanocarbons	N‐doping C−C sp3 defects	Pyrolysis and etching	280	[124]
K‐ion	MoS2_(1–*x*)_ *Se*_2*x*_ alloys	Sulfur/selenium vacancies	Alloying reaction	517	[125]
Al‐ion	Porous 3D graphene foam	Highly porous	Plasma technology	148	[126]
Li‐ion	F‐doped graphene	Edge/surface	Liquid exfoliation	>1000	[102]
Li‐ion	Fluorographene	Edge/surface	Solvothermally exfoliated	775	[103]
Li‐ion	Halogenated graphene	Edge/surface	Ball‐milling	1783.6	[106]
Li‐ion	Fluorinated graphene	Edge/surface	Ball‐milling	1778.1	[108]
Li‐ion	N‐doped graphene	Edge/surface	CVD	0.25 mAh cm^−2^	[127]
Li‐ion	N‐doped graphene	Edge/surface	Heat treatment	>800	[128]
Li‐ion	N‐ & F co‐doped graphene	Edge/surface	Hydrothermal	1894	[129]
Li‐ion	3D M_*x*_ MnO_2_	Edge/surface	Self‐assembly	155	[130]
	(M=Li, Na, K, Co and Mg)				
Li‐ion	Hydrogenated V_2_O_5_	Edge/surface	H2 thermal treatment	259	[131]
Li‐ion	Defect‐rich MoS_2_	Edge/surface	Hydrothermal	1179	[132]
K‐ion	VOPO_4_‐graphene	vdW heterostructure	solution‐phase	160	[133]
			self‐assembly strategy		
K‐ion	F‐doped graphene	Edge/surface	solid‐state synthetic	165.9	[134]
Li‐ion	defective NiB_6_	Vacancy	Theoretical	1301.61	[135]
Na‐ion	defective NiB_6_	Vacancy	Theoretical	1301.61	[135]
K‐ion	defective NiB_6_	Vacancy	Theoretical	1301.61	[135]
Li‐ion	BC_3_	S−W defect	Theoretical	1287	[136]
Li‐ion	TMPS_3_	Vacancy	Theoretical	441.65–484.34	[137]
Li‐ion	defective V_2_C	Vacancy/surface	Theoretical	301.12	[38]
Na‐ion	defective V_2_C	Vacancy/surface	Theoretical	301.12	[38]
Li‐ion	Fe_3_C@DRC	Doping	Sol‐gel	215	[138]
Li‐ion	Defective C_3_N	Doping	Theoretical	534.42	[139]
Li‐ion	Phosphorene	Doping	Theoretical	800	[140]

* TMPS_3_‐transition metal phosphorus trisulfides (TM=Mn, Fe, Co, Ni)

**Figure 5:**
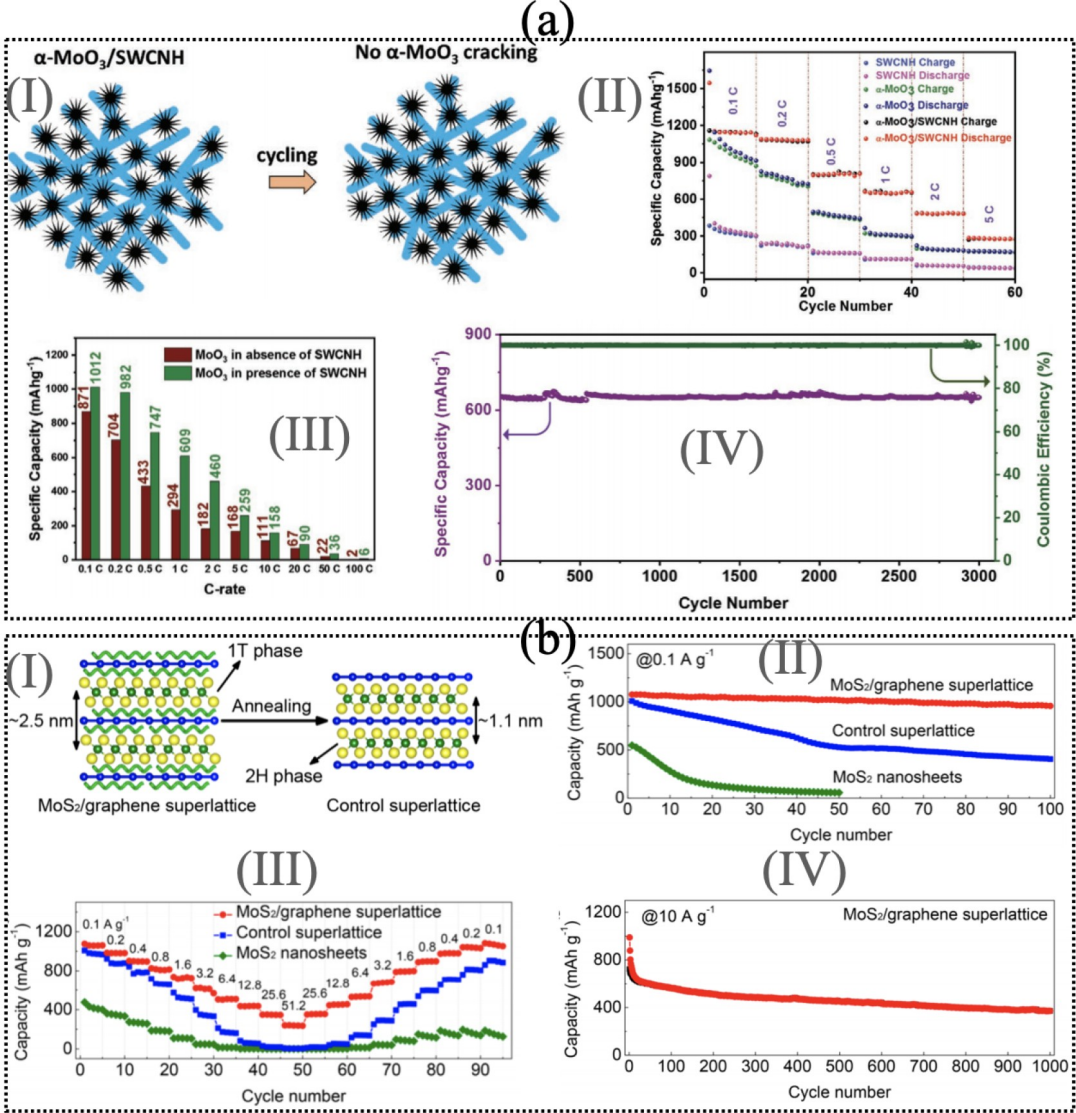
(a) (I) Schematic illustration of the functionality of *α*‐MoO_3_/SWCNH composite material, (II) charge–discharge profile of *α*‐MoO_3_/SWCNH hybrid structure at different C‐rates between 0.1 to 5 C, (III) specific capacity of *α*‐MoO_3_ in the presence and absence of SWCNHs at various C‐rates, and (IV) cycling performance and corresponding Coulombic efficiency of *α*‐MoO_3_/SWCNH hybrid structure at 1 C. Reprinted with permission from Ref. [162]. (b)(I) Schematic representations of the formation of the 2H phase and decreased interlayer spacing of MoS_2_ during the annealing process for the control superlattice, (II) cycling performances of the MoS_2_/graphene superlattice, control superlattice, and MoS_2_ nanostructure at 0.1 A g^−1^ for 100 cycles, (III) rate capability of the MoS_2_/graphene superlattice, control superlattice, and MoS_2_ nanostructure and (IV) long‐term cycling stability of the MoS_2_/graphene superlattice at 10 A g^−1^ for 1000 cycles. Reprinted with permission from Ref. [163].

According to Xiao et al.,^[164]^ sucessessfully synthesized the MoS_2_/PEO composite in the ratio of 0.05. After that MoS_2_/PEO composite was used for Li‐ion battery which has high capacity of 1000 mAh g^−1^. Furthermore, another composite material MoS_2_ with disordered graphene‐like layers have superior capacity of 700 mAh g^−1^ at the rate of 50 C.^[165]^ Teng et al.^[166]^ reported that the MoS_2_/graphene composite for Li‐ion battery reached a capacity of 1077 mAh g^−1^ after 150 cycles at a current density of 100 mA g^−1^ and still maintained 907 mAh g^−1^ over 400 cycles at 1.0 A g^−1^. The significant changes was observed in case of WS_2_/rGO displayed high reversible capacity of 697.7 mAh g^−1^ for LIBs after 100 cycles at a current density of 100 mA g^−1^, while pristine WS_2_ nanosheet exhibited a capacity of 88.5 mAh g^−1^.^[167]^ Recently it has been synthesized the $\alpha -MoO_{3}$ /SWCNH hybrid nanomaterials^[162]^ as presented in Figure 5 a(I). $\alpha -MoO_{3}/SWCNH$ hybrid nanomaterials is reported for Li‐ion battery and displayed the reversible capacities of 1132 mAh g^−1^ at 0.1 C which is significantly higher than its isolated nanomaterials (see Figure 5a(II)). Figure 5a(III) displayed the presence and absence of interfacial effect of SWCNH on the surface of $\alpha -MoO_{3}$ to see the exact changes in the electrochemical performance of Li‐ion battery. It was also seen that the $\alpha -MoO_{3}/SWCNH$ hybrid nanomaterials have long‐term cycling performance after 3000 cycles with Coulombic efficiency of 99% (Figure 5a(IV)). Also, rGO/MoO_3_ composite nanomaterial exhibited high reversible capacity of 900 mAh g^−1^ at the rate of 0.1 C after 100 cycles.^[168]^ Another group reported that the porous MoO_3_/MWCNT composite nanomaterial have reversible capacity of 1350 mAh g^−1^ at 500 mA g^−1^ after 300 cycles.^[169]^ Xiong et al. synthesized the MoS_2_/graphene composite nanomaterial with control superlattice (see Figure 5b(I)) for Na‐ion battery.^[163]^ It shows the initial discharge and charge capacities of 2220 and 1100 mAh g^−1^ at 0.1 A g^−1^, respectively, which is relatively higher than its isolated nanomaterials as presented in Figure 5b(II). Figure 5b(III) displayed the capacity at different current density. It can be notice that the MoS_2_/graphene hybrid nanomaterials exhibited a specific capacity of 240 mAh g^−1^ at high current density of A g^−1^. Also MoS_2_/graphene hybrid nanomaterials displayed a high retention stability after 1000 cycles at 10 A g^−1^ presented in Figure 5b(IV). A very recent report shows a very high initial discharge‐charge capacity of 1409.4 mAh g^−1^ and 968.9 mAh g^−1^ for N‐SiO_*x*_/C/GF‐4 composite material for Li‐ion battery.^[170]^ It also displays an better cycling performance of 525.2 mAh g^−1^ over 500 cycles at 1 A g^−1^.

We conclude that the approach of modifications of the structure, including the regulation of the thickness, handling of porosity and modulating the morphology, provides excellent opportunities to exploit to the maximum the merits of a structure 2D nanolayers. A refining the surface morphology and structure of hierarchical nanostructures, trends autoaggregation of nano 2D sheets can be greatly reduced and the electrochemical reaction between the active materials and electrolyte can be accelerated with high kinetic. Hierarchical nanostructures 3D with 2D components have the characteristics of the most active sites, a good contact interface and changes shameful volume and, therefore, help improve the mobility and convenience of next generation metal‐ions batteries.

## 4 Summary and outlook

In the present mini‐review, we focus on recent research and understanding of the role of defects chemistry on 2D materials in rechargeable battery for electrode materials. Due to the various defects included in 2D materials for examples heteroatomic doping, intrinsic defects, vacancy defects, topological defects, line defects, etc., which will alter the atomic structure and charge distribution, thus improving the diffusion of ions and electron transfer, the defective 2D electrode material is one of the superior materials for the battery. Specifically, the introduction of defects into nanomaterials can not only increase the storage site foreign ions and effectively improve the battery capacity. In addition, several studies have reported that the initiative of defects produces a enormous number of active sites, which can effectively improve the electrochemical phase transitions and promote reaction kinetics. Furthermore, the introduction of defects in the electrode materials can also accomplish structural stability and high flexibility when foreign ions insertion and extraction. The aim of this mini review is to highlight the positive role of engineering defects in optimizing the electrode materials to achieve sustainable improvements for applications of rechargeable batteries. The characterization of some electrode materials via engineering defects, the recent reports in the literature also abbreviated in Table 1. Some of the specific challenges are presented to further promote the application of defective electrodes. It is also very important to control the characterization of several defects in electrode materials and clarify the specific contribution of each defect to the performance of electrode materials. In addition, it is an important research direction to study the dynamic evolution of defective materials electrode during discharge/charge battery using the electrochemical technique of in situ spectroscopy. It is of great importance in understanding the surface chemistry of electrode materials and the reaction mechanism could be on‐site monitoring of electrode materials, especially those with rich and highly active defects. The intermediate generated at the surface of the electrode materials is detected by the tracking of the dynamic changes of the electrode materials in the charge and discharge process. Using the modelling of theoretical simulations, to explore the active site of the reaction and revealing mechanism of degradation, thus providing the solution to the stability and other problems for the batteries.

Additionally, along with the increasing research fever of 2D materials as electrode materials for rechargeable battery, many researchers found that the defects chemistry on the surface of 2D materials might induce positive effects in the electrochemical reaction and more especially might overcome a major battery‐related issue of structural instability of the electrode materials in the electrochemical process, which leads to low electrochemical efficiency, namely rapid‐capacitance as well as a high‐voltage attenuation. Since then, several researches has demonstrated the fact that introducing defect chemistry on 2D materials electrode provide greater structural stability during the extraction/insertion of metal‐ions and thus improving the electrochemical reversibility.

In summary, the active part of the defect chemistry 2D materials in battery electrode materials are systematically summarized in this mini review. Although there are some challenges to explore completely still, it is strongly recommended to accelerate research and development of technologies related to overcome existing shortcomings. In general, progress has been made in implementing engineering defects rechargeable batteries. We consider that continuous attention from countries around the world, defective electrode materials can also be applied in other fields of storage and electrochemical energy conversion. Therefore, it is believed that these strategies effective in improving lithium storage of 2D defective nanomaterials are good benchmarks for researchers and scientists in related fields of 2D nanomaterials, chemistry and nanotechnology, which hope to develop rechargeable batteries superior next generation.

## Conflict of interest

The authors declare no conflict of interest.

## Biographical Information

*Nabil Khossossi received his M.Sc degree in Mathematical & Theoretical Physics from the Department of Physics at Mohamed V university, Rabat. Then, in 2018 he joined Material Physics and System Modeling laboratory at Department of Physics, Moulay Ismail University, Meknes, Morocco, as Ph.D. candidate under the supervision of Prof. Ismail Essaoudi & Prof. Abdelmajid Ainane. He is currently a visiting research student under the supervision of Prof. Rajeev Ahuja at Materials Theory Group, Department of Physics and Astronomy, Uppsala University, Sweden. His PhD work focus on unifying energy harvesting through 2D materials modelling, including energy conversion and storage applications. He is currently interested in DFT‐based multi‐scale modeling for the understanding and design of new energy materials and developing machine learning approach for 2D materials electrodes*.

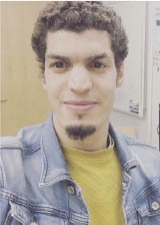

## Biographical Information

*Deobrat Singh is a postdoctoral researcher at Materials Theory Group, Department of Physics and Astronomy, Uppsala University, Sweden under the supervision of Prof. Rajeev Ahuja. . His research interest spans over a broad range, wherein he employs diverse computational tools to explore various significant scientific problems related to nanomaterials, catalysis, sensors, molecular electronics, materials for energy storage including solar cells, perovskites, batteries, thermoelectric devices and other applications of novel two‐dimensional monolayer/multilayer materials. He has published more than 67 scientific papers in peer‐reviwied journals (with a total citations ∼560; h‐index = 11) covering these topics. Recently he recieved the young achiever award in 2019*.

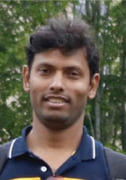

## Biographical Information

*Abdelmajid Ainane is a full time Professor in the Department of physics, Faculty of Sciences at Moulay Ismail University, Meknes, Morocco, Head of the magnetism and systems modeling group (2MS). The Group is composed of 18 people. In his scientific career, he has supervised around 20 PhD students and he is one of the good researchers of the Moroccan University. Ainane's research covers a wide range of topics in nano‐structured materials, condensed matter Physics and materials Science. His current research is focused on four major areas: 2D Materials & Energy storage and Batteries, Perovskites Solar Cells, Magnetism and Nanomagnetism, Spintronics. Ainane is the author of more than 140 papers and a referee for several scientific journals. Ainane received the “NIKOLA TESLA” prize in 2015. Since November 2019, Ainane has Headed the Physics Department of Moulay Ismail University made up of 70 academics and 10 technicians/engineers*.

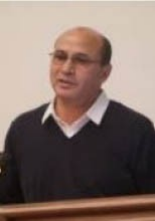

## Biographical Information

*Rajeev Ahuja is professor of Material Theory at Uppsala University and one of the most highly cited researchers in Sweden under 55. He has published 910 scientific papers in peer reviewed journals (H‐Index 83 (Google Scholar), i‐10‐index 545 & citations more than 32000). Ahuja has recently elected (Sept.2019) APS‐Fellow by American Physical Society (APS), USA, Appointed in the advisory Board of journal, Journal of Materials Chemistry A from Royal Society of Chemistry (England) & awarded Beller Lectureship for the APS March Meeting 2017, in New Orleans, USA. He been awarded the Wallmark prize for 2011 from KVA (Royal Swedish Academy of Sciences), and has previously received the Eder Lilly and Sven Thureus prize and the Benzelius prize from Royal Society of Sciences (KVS). Ahuja is an elected member of the Swedish Royal Society of Sciences and served on the board of the European High Pressure Research Group as well as of the executive board of the International Association for the Advancement of High Pressure Science and Technology. Ahuja has supervised 30 PhD students and more than 35 postdocs*.